# Internet Gaming Disorder, Risky Online Behaviour, and Mental Health in Hong Kong Adolescents: The Beneficial Role of Psychological Resilience

**DOI:** 10.3389/fpsyt.2021.722353

**Published:** 2021-10-15

**Authors:** Yvonne Yin-yau Tsui, Cecilia Cheng

**Affiliations:** Department of Psychology, The University of Hong Kong, Hong Kong, Hong Kong, SAR China

**Keywords:** problemic gaming, gaming addiction, online risk, psychological resilience, Chinese, adolescence, psychological well-being

## Abstract

In the present cyber age, Internet gaming disorder (IGD) and risky online behaviour are prevalent, and adolescents are especially vulnerable to such emergent problems. Few studies have explored the protective factors that mitigate harm caused by IGD and various common risky online behaviours. This study examined the prevalence of IGD and risky online behaviour, their hypothesised associations with depressive symptoms, and the beneficial role of psychological resilience as an underlying psychological mechanism. The participants included 1,099 Chinese junior secondary school students (33% boys, mean age = 13.5 years, age range = 10–17 years) who completed a battery of validated self-report questionnaires at their schools. The results revealed that 4% of the participants were at high risk of IGD and 6% were at an overall risk level of IGD. Depressive symptoms were positively associated with IGD and risky online behaviour, and psychological resilience mediated both of these associations. These results imply that clinicians and teachers should incorporate psychological resilience training into intervention approaches to mitigate IGD and risky online behaviour.

## Introduction

### Background

Digital technologies are widespread in many aspects of contemporary life, resulting in abundant new forms of social interaction in the cyberspace. Research on the mental health problems associated with Internet gaming disorder (IGD) has grown over the last decade. Adolescents are susceptible to a range of mental health problems associated with prolonged Internet gaming (1–3), including depression, social anxiety, and loneliness (4–6). Online gaming and risky online behaviour have been used as methods to cope with psychological difficulties caused by real-life problems that generate depression and loneliness (7). Psychological resilience may serve as an essential element to mitigate these psychological risks and challenges during puberty. To address these timely but unexplored issues, this study investigated how psychological resilience would mediate the association of depressive symptoms with both IGD and risky online behaviour. The findings advance the understanding of emergent issues that enable the development of preventive strategies for IGD and risky online behaviour in adolescents.

According to the 11th edition of the International Classification of Diseases (ICD-11), gaming disorder is defined as:

*persistent or recurrent gaming behaviour characterised by an impaired control over gaming, increasing priority given to gaming over other activities to the extent that gaming takes precedence over other interests and daily activities and continuation of gaming despite the occurrence of negative consequences*.

IGD is also classified as a condition in the fifth edition of the Diagnostic and Statistical Manual of Mental Disorders (DSM-5). It includes nine main criteria that resemble the conceptualisation of addictive behaviours, such as pathological gambling and substance use (8). The criteria include (1) obsession; (2) withdrawal; (3) tolerance building; (4) impaired control; (5) loss of interest in other activities and hobbies; (6) continued play, despite knowing the consequence of its impact on life; (7) a tendency to lie about the frequency of gaming; (8) a desire to escape from psychological problems; and (9) loss of opportunity in work or study or significant relationships (9). Although IGD is conceptually similar to Internet addiction, the two constructs are distinct because empirical evidence indicates that they have different correlates (10). Specifically, Internet addiction is positively associated with online chatting, gaming, and social networking while IGD is positively associated only with online gaming (11). The global prevalence of IGD is around 3% (12). In Asia, the prevalence of IGD among adolescents is estimated to be 5%, and the prevalence is seven times higher among male adolescents than female adolescents (13, 14).

Risky online behaviour refers to hazardous online actions or activities that may imperil users' daily lives (15). Common risky online behaviours include meeting strangers, sharing personal data, exposure to violence-related online resources, cyberpornography, initiating cyberbullying, and using abusive language online (16, 17). The prevalence of cyberbullying ranges between 20 and 40% in the majority of studies (18). Studies have shown a high prevalence rate of 57% for cyberbullying in China (19), and 15% of Chinese students reported exposure to moderate-to-severe violence while playing online games (20). In another sample of American adolescents aged 10 to 17, 9% reported having met online strangers in person and 14% reported having watched cyberpornography, with one third of them reporting psychological distress (21).

Psychological resilience is a construct that reflects a person's ability to endure and cope with adverse psychological outcomes in stressful encounters and to adjust and overcome setbacks (22). Accordingly, the protective model of psychological resilience defines this construct as “positive patterns of adaptation in the context of risks or adversity,” which is a complex and provocative area in psychosocial development (23) (p. 24). From the perspective of positive psychology, an individual's strength is similar to their psychological resilience; it not only helps the individual to flourish but also protects them from developing addictive behaviours (24). Psychological resilience comprises three major protective mechanisms pertaining to individual capacities (e.g., personal skills, social skills), interpersonal relations (e.g., peer support, physical and psychological caregiving), and the availability of community resources and opportunities (e.g., spirituality, culture, education), respectively (25, 26). These protective mechanisms strengthen a person's ability to handle an array of environmental and personal problems, such as daily stressors and interpersonal conflict (27).

### Association of Depression With IGD and Risky Online Behaviour

In the literature, depression has been shown to be a predisposing factor for Internet-related behaviours (28–30). Some studies have shown that depression is related to the initiation and persistence of IGD in adolescents (31–33). A longitudinal study further illustrated a possible bidirectional association between IGD and depressive symptom that share similar risk factors (3), such as low educational achievement and poor self-regulation (34–36). Specifically, individuals with depression tend to use the Internet excessively as a way to self-medicate, which is an avoidant coping strategy that leaves real-life problems intact rather than solving them (37). In addition, IGD itself may elicit more depressive symptoms due to disruption of real-life social interactions (38), resulting in social isolation and interpersonal problems that become a vicious cycle (3, 39). According to the neurocognitive model (40), problematic online gaming experiences alter gamers' brain functions and cognitive processes, such as gaining a sense of reward and diminished executive function in fulfilling important life responsibilities, which can elicit a sense of failure and enhance the likelihood of developing depressive symptoms.

Individuals with psychological difficulties are more likely to engage in risky online behaviours, including cyberpornography (41), which are in turn associated with low self-esteem and depression (42). In a longitudinal study, adolescents who experienced cyberbullying victimisation rated their coping strategies as helpless, which was associated with depressive symptoms (43). Adolescents with low self-esteem and high depressive symptoms are more likely to play violent video games (44). In light of this literature review, depressive symptoms may play a role in both IGD and risky online behaviour. Thus, the following hypotheses were formulated and tested.

H1a: Depressive symptoms are positively related to IGD.
H1b: Depressive symptoms are positively related to risky online behaviour.

### The Mediating Role of Psychological Resilience

Psychological resilience is a vital factor that protects adolescents from experiencing harm while engaging in online activities, especially among those with Internet addiction. Greater psychological resilience alleviates psychological impacts associated with Internet addiction and online risk exposure (45, 46). Studies have also shown that individuals with lower psychological resilience are more likely to immerse themselves in the virtual network (47). Such findings highlight the possible role of psychological resilience in preventing the development of IGD and attenuating mental health problems that are prevalent in today's society. In addition, a recent study indicates that individuals with IGD generally have lower levels of psychological resilience compared with individuals without IGD (48). IGD together with low psychological resilience engender symptoms of depression and anxiety (38, 49).

Scant research efforts have been expended to specifically examine the association between psychological resilience and risky online behaviour. Research has indicated that adolescents with a low level of psychological resilience tend to experience at least one psychological disruption after being exposed to one or more online risks (50). Those with a higher level of psychological resilience, however, are less likely to be disrupted by risks encountered while engaging in online activities. Moreover, adolescents with a higher level of psychological resilience (vs. lower) are more likely to recognise online risks, deflect unusual approaches, and have adequate confidence to protect and inform others when necessary (51).

Considering the aforementioned theories and findings that imply the role of psychological resilience in IGD and risky online behaviour, psychological resilience is proposed to mediate the hypothesised association of depressive symptoms with both IGD and risky online behaviour. Hence, we tested the following hypotheses:

H2a: Psychological resilience mediates the association between depressive symptoms and IGD.
H2b: Psychological resilience mediates the association between depressive symptoms and risky online behaviour.

### Moderated Mediation Effect of Sex

This study further examined sex differences, because previous studies have documented that women are more likely to experience depression than men (52), and women generally seek social support to relieve psychological distress more than men (53). Moreover, research has shown that men (vs. women) are at higher risk of developing IGD (54) due to a lack of help-seeking tendencies and indulgence in online gaming as an avoidant coping strategy, both of which increase the likelihood of depressive symptoms (55). Considering the greater frequency of men (vs. women) playing online gaming and watching cyberpornography (56), the magnitude of the association between risky online behaviour and depressive symptoms also tends to vary by sex. In light of these findings, we propose that sex differences influence the magnitude of the association of depressive symptoms with both IGD and risky online behaviour. Thus, the following hypotheses were tested:

H3a: Sex moderates the indirect effect of depressive symptoms on IGD.
H3b: Sex moderates the indirect effect of depressive symptoms on risky online behaviour.

In summary, this study examined psychological resilience as a mechanism underlying the association between problematic online behaviours and depressive symptoms. Although some studies have examined individuals with Internet addiction, research on the interplay between risk factors and protective factors in both IGD and risky online behaviour in adolescents remains scarce in terms of adverse psychological outcomes and social impact. As mentioned at the outset, IGD and Internet addiction are conceptually similar yet they are distinct (10), with each having its unique set of psychological correlates. Hence, more empirical work needs to examine IGD, which was recently classified as a mental disorder in the ICD-11. In addition, we expanded the scope to examine not only specific Internet-related disorders but also an array of risky online behaviours.

To fill the knowledge gaps, this study investigated depressive symptoms as risk factors for both IGD and risky online behaviour, and more importantly, it examined the hypothesised mediating role of psychological resilience on depressive symptoms and these emerging problems in the cyber era. To test these hypotheses, we conducted a cross-sectional survey in a sample of secondary school students. To the best of our knowledge, this study is the first to unveil the mechanisms of psychological resilience underlying the association of depressive symptoms with both IGD and risky online behaviour. The findings have practical implications for the design of prevention and intervention programmes aimed at mitigating these emerging problems in the cyber age.

## Methodology

### Participants and Procedures

The participants included 1,099 Chinese students from secondary school Year 1–4. The sample was recruited from three secondary schools subsidised by government funding. Government-subsidised school is chosen because the most common type that constitutes 90% of all secondary schools in Hong Kong (57). The schools were randomly selected from three major regions of Hong Kong. Most participants (67%) were female, and the age range of the participants was 10–17 years old, with a mean age of 13.5 years (*SD* = 1.17).

The human research ethics committee of the authors' University approved the research protocol before the start of the study. Passive consent was obtained from parents or legal guardians, who received an invitation letter informing them that they could refuse their child's participation without any adverse consequences by returning the consent form to their teachers. Before the study began, the student participants were invited to give written informed consent, with the explanations by a trained research assistant in the classroom. They were told that they had the right to refuse to participate in the study and to withdraw from the study at any time without any negative consequences. The participants had 20–25 min to complete the questionnaires with 125 items. Participation in this study was anonymous and voluntary, and confidentiality of personal data was ensured.

The data collection sessions were conducted by trained research assistants to ensure that the students fully understood the instructions and questions. Under the assistants' guidance, most of the students completed all the items in the questionnaires, with the exception of four participants (0.4%). As the four participants had omitted more than half of the items, their data were discarded. The final sample thus consisted of 1,095 participants.

### Measures

#### Psychological Resilience

To assess psychological resilience, the self-report version of the Child and Youth Resilience Measure-28 (26) was used because it has been previously validated among Chinese youngsters (58). The 28 items measure three key constructs: individual factors (e.g., personal skills, peer support, social skills), relationship with the primary caregiver (e.g., physical and psychological caregiving), and contextual components that facilitate a sense of belonging (e.g., spirituality, culture, education). The participants rated each item on a 5-point Likert scale ranging from *not a lot* (1) to *a lot* (5). This scale was internally consistent in this study (Cronbach's alpha = 0.93).

#### IGD

The self-report of the adapted version of the Korean Internet Addiction Proneness Scale (59) was adopted because it is the only scale that has been developed and validated for Asian adolescents in case classification (60). For all the 15 items of the validated adapted version, the term “Internet use” was replaced with “video game playing.” Specifically, the participants who scored ≤ 40 were classified as “average gamers,” those who scored between 41 and 43 were classified as “at-risk gamers,” and those who scored ≥ 44 were classified as “high-risk gamers.” This classification was validated among Hong Kong individuals (61). The 4-point Likert scale ranged from *strongly disagree* (1) to *strongly agree* (4). The measure had good reliability in this study (Cronbach's alpha = 0.76).

#### Risky Online Behaviour

Given that a comprehensive measurement tool to assess risky online behaviour was unavailable, a questionnaire with 31 items was compiled by extracting relevant items from the Risky Online Behaviour Inventory (62) and the survey protocol constructed by Livingstone et al. (63). The combined measure assessed six common types of risky online behaviour, including cyberbullying perpetration, cyberbullying victimisation, exposure to online pornography, exposure to online violent content, meeting online strangers, and sharing personal data online. The participants rated the items using a 5-point Likert scale (*never, rarely, sometimes, very often*, and *always*). The scale had high internal consistency in this study (Cronbach's alpha = 0.89).

#### Depressive Symptoms

The Chinese version of the standardised Centre for Epidemiologic Studies Depression for Children Scale (64) was adopted to assess the tendency toward depression because it has been validated in both Western (65) and Chinese (66) contexts. The 4-point Likert scale ranged from 1 (*rarely or none of the time*) to 4 (*all the time*). The 10-item scale has been adopted as a screening measurement tool with higher cut-off scores indicating greater symptomatology (67, 68). The scale was found to be reliable in this study (Cronbach's alpha = 0.86).

#### Data Analysis Approach

For the preliminary analysis, a multivariate analysis of variance (MANOVA) was performed to examine differences among high-risk, at-risk, and regular gamer groups. The effect size was evaluated using partial η^2^. Pearson's correlation was then used to indicate the magnitude of the association among the study variables. The Bonferroni correction method was adopted to reduce Type I error, and the adjusted alpha level was 0.007 (i.e., 0.05/7).

For the main analysis, the hypothesised mediation effects were tested using PROCESS macro (69), a statistical technique widely regarded as the standard method to test mediation and moderation effects (70). The bootstrap method was adopted to provide more reliable estimates of these hypothesised effects and to minimise Type I error. Following Hayes' (71) recommendations, the data were resampled 5,000 times. PROCESS Model 4 was applied for the mediation analysis, and PROCESS Model 59 was used for the moderated mediation analysis, the latter of which tested the moderating effect of sex in each mediation model [see (71) for details of these models]. In these analyses, the coefficients of both direct and indirect effects, as well as their 95% confidence intervals (CIs), were reported, and a significant effect was indicated when the 95% CI excluded 0. All of the data analyses were conducted using SPSS version 23.

## Results

### Risk-Level Classifications

In the sample, 4% of the participants were at high risk of IGD, 6% were at an overall risk level, and 90% were classified as average gamers. Table 1 summarises the descriptive statistics for these three IGD groups and for the pooled sample.

**Table 1:** Descriptive statistics of study variables by IGD risk group and pooled sample.

	**High-risk gamers** ***n*** **=** **43**		**At-risk gamers** ***n*** **=** **65**		**Average gamers** ***n*** **=** **987**		**Pooled sample** ***n*** **=** **1,095**		
**Measure**	***M***	***SD***	***M***	***SD***	***M***	***SD***	***M***	***SD***	**Cronbach's α**
**Demographics**									
Age	13.56	1.12	13.13	0.95	13.22	1.18	13.23	1.17	
**Internet Usage**									
Hours spending online	5.50	3.68	3.92	2.48	3.47	2.61	3.58	2.68	
Hours spending on online gaming	4.26	3.41	2.66	2.03	1.79	1.85	1.95	2.05	
Hours spending on SNS	2.39	3.17	1.92	1.79	1.73	1.82	1.75	1.87	
**Psychological well-being**									
Psychological Resilience	95.06	16.52	97.96	14.57	103.03	14.85	102.29	15.22	0.93
Depression	14.88	6.41	11.06	5.53	8.48	5.78	8.91	5.97	0.86
Risky online behaviour	11.44	2.46	9.60	1.73	9.03	2.29	9.17	2.33	0.89
Sharing personal data	1.64	0.67	1.45	0.50	1.42	0.57	1.43	0.58	
Meeting strangers	2.57	0.90	1.93	0.62	1.83	0.73	1.86	0.74	
Violence	2.43	0.94	2.18	0.78	1.82	0.75	1.88	0.78	
Bullying	1.47	0.55	1.28	0.34	1.27	0.39	1.28	0.40	
Pornography	1.56	0.67	1.39	0.45	1.32	0.54	1.35	0.55	
Being bullied	1.78	0.65	1.50	0.46	1.40	0.50	1.43	0.51	

*SNS, social networking site*.

For psychological resilience, there was a significant difference among groups with distinct risk levels of IGD, *F*_(2,895)_ = 7.209, *p* = 0.001; effect size = 0.16. *Post hoc* Tukey's test revealed that the average gamer group reported a greater level of psychological resilience compared with the high-risk gamer group (*p* = 0.044) and the at-risk gamer group (*p* = 0.006). However, there was no significant difference between the high- and at-risk gamer groups in terms of the level of psychological resilience (*p* = 0.649). For depressive symptoms, the high-risk gamer group reported a higher score than the average gamer group (*p* < 0.0001).

### Sex Differences

Table 2 summarises the descriptive statistics and interrelationships among the study variables by sex. There was no difference in psychological resilience between the sexes. However, the male participants reported higher levels of both IGD and risky online behaviour than their female counterparts. The difference in the time spent on Internet gaming was also prominent, with the male participants spending more time than the female participants. Similarly, exposure to violent content was more common in the male participants than in the female participants. In contrast, depressive symptoms were more commonly experienced in the female participants than in the male participants.

**Table 2:** Sex differences in study variables.

	**Mean (SD)**		***t* (*p*)**	
	**Female^a^**	**Male^b^**		**Cohen's *d***
Psychological Resilience	102.255 (14.611)	102.333 (16.462)	−0.078 (0.938)	0.005
Internet Gaming Disorder	33.011 (6.118)	34.194 (5.590)	−3.095 (0.002)^***^	0.202
Risky online behaviour	8.866 (2.172)	9.863 (2.553)	−6.580 (0.000)^***^	0.421
Share data	1.396 (0.524)	1.517 (0.673)	−3.317 (0.001)^***^	0.201
Meeting strangers	1.821 (0.721)	1.943 (0.777)	−2.597 (0.010)^**^	0.163
Violence	1.667 (0.668)	2.297 (0.821)	−13.751 (0.000)^***^	0.842
Bullying	1.270 (0.369)	1.306 (0.462)	−1.375 (0.169)	0.064
Pornography	1.311 (0.490)	1.418 (0.658)	−3.082 (0.002)^***^	0.185
Being bullied	1.416 (0.486)	1.447 (0.569)	−0.961 (0.337)	0.059
Loneliness	34.710 (10.173)	33.159 (10.006)	2.350 (0.019)^*^	0.154
Depression	9.645 (6.213)	7.409 (5.121)	5.892 (0.000)^***^	0.393
Online hours	3.553 (2.568)	3.723 (3.523)	−0.928 (0.354)	0.057
Internet gaming hours	1.381 (1.728)	3.197 (3.229)	−12.137 (0.000)^***^	0.701
Social networking site hours	1.850 (1.815)	1.672 (3.836)	1.048 (0.295)	0.059

* *p <0.05*,

** *p <0.01*,

*** *p <0.001*.

a *n = 755*;

b *n = 372*.

### The Mediating Role of Psychological Resilience

**Figure 1:**
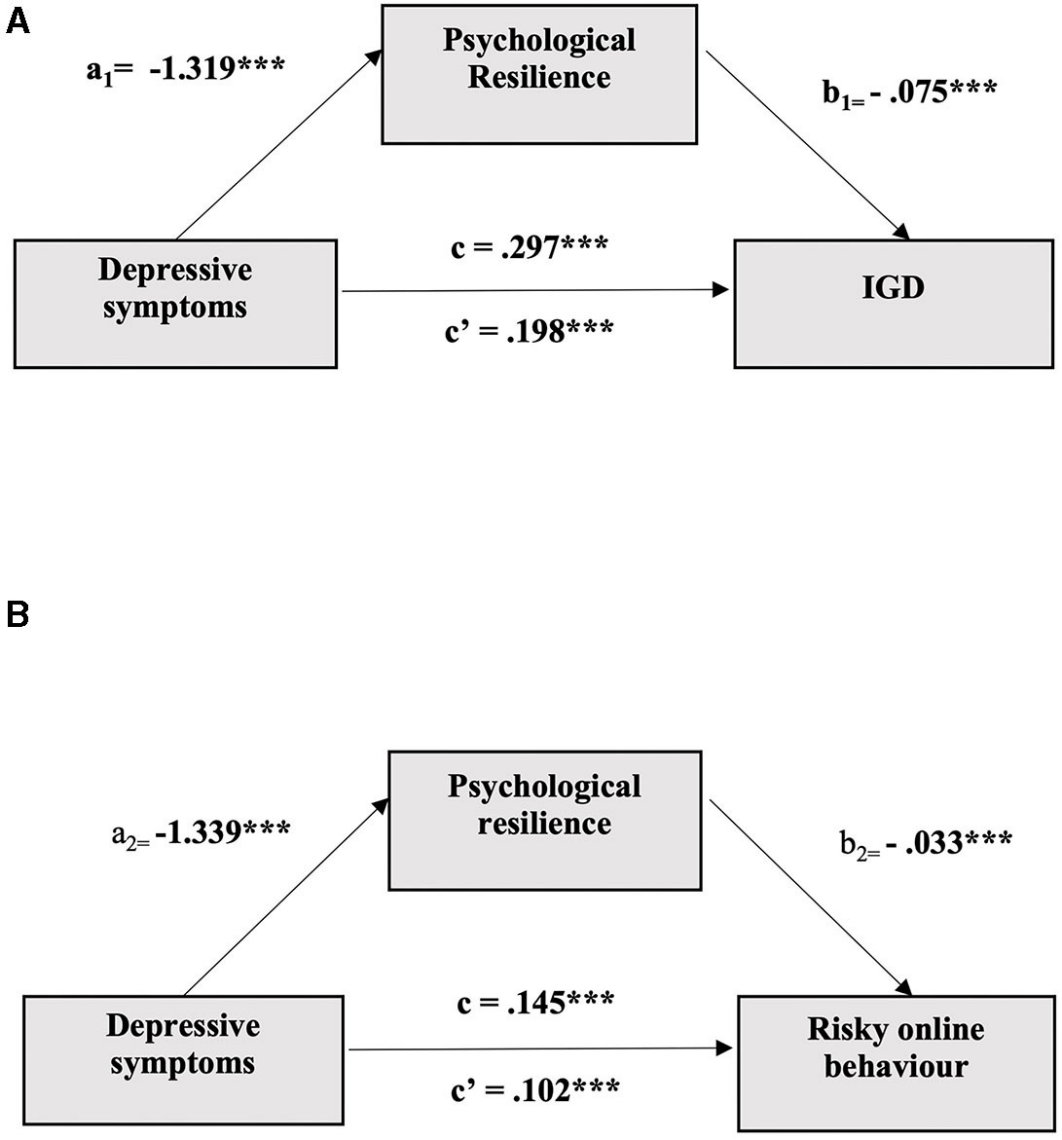
**(A)** Mediation Model A with psychological resilience mediating the association between depressive symptoms and IGD (***p < 0.001. IGD, Internet gaming disorder; a1, b1, c, and c′ represent each path coefficient; c′ = direct effect of depressive symptoms on IGD with controlling mediating effect; c = total effect of depressive symptoms without controlling mediating effect; all values are unstandardized). **(B)** Mediation Model B with psychological resilience mediating the association between depressive symptoms and risky online behaviour (***p < 0.001. a2, b2, c, and c′ represent each path coefficient; c′ =direct effect of depressive symptoms on risky online behaviour with controlling mediating effect; c = total effect of depressive symptoms without controlling mediating effect; all values are unstandardized).

The hypothesised mediating effect of psychological resilience was tested in two models, one with IGD (Model A) and the other with risky online behaviour (Model B) as the criterion variable. The results of both models are summarised in Table 3, and the mediation models are depicted in Figures 1A,B.

**Table 3:** Direct and Indirect Effects of mediation models A (criterion variable = IGD) and B (criterion variable = risky online behaviour).

	**β**	**Boot SE**	***t***	**Boot LLCI**	**Boot ULCI**
**Model A**					
Total effect of Depression → IGD	0.297	0.031	9.618	0.237	0.358
Direct effect (c′)	0.198	0.036	5.538	0.128	0.268
Total indirect effect	0.099	0.020		0.061	0.140
Depression → psychological resilience → IGD	0.099	0.020		0.061	0.139
**Model B**					
Total effect of Depression → Risky online behaviour	0.145	0.012	12.546	0.122	0.168
Direct effect (c′)	0.102	0.013	7.613	0.075	0.128
Total indirect effect	0.044	0.007		0.029	0.058
Depression → psychological resilience → Risky online behaviour	0.113	0.019		0.077	0.151

*IGD, Internet Gaming Disorder; LLCI, Lower limit confidence interval; ULCI, Upper limit confidence interval*.

The upper panel of Table 3 shows that the direct effect of depressive symptoms on IGD was significant, and the indirect effect of these factors through psychological resilience was also significant (*p*'s <0.0001). As shown in Figure 1A, the participants with more (vs. less) depressive symptoms tended to report a lower level of psychological resilience, which in turn was related to a higher score for IGD (*p*'s <0.0001).

A similar pattern was observed for the criterion variable of risky online behaviour. The lower panel of Table 3 reveals a significant direct effect of depressive symptoms on risky online behaviour, and the indirect effect of these factors through psychological resilience was also significant (*p*'s <0.0001). Referring to Figure 1B, the participants with more depressive symptoms tended to report a lower level of psychological resilience, which in turn was related to a higher level of risky online behaviour (*p*'s <0.0001). Both sets of findings were in line with our hypotheses.

### Moderated Mediation Effect of Sex

The results revealed no significant moderation effect of sex in the above mediation models. For the criterion variable of IGD (Model A), the hypothesised moderated mediation effect through psychological resilience was 0.020 (95% CI = −0.070 to 0.117). As the CI contained 0, this effect was not significant.

The results were similar for the criterion variable of risky online behaviour. The hypothesised moderated mediation effect through psychological resilience was 0.029 (95% CI = −0.018 to 0.048). The CI included 0, indicating a non-significant effect. In short, both sets of results failed to support the moderated mediation effect of sex.

## Discussion

This study contributes to the literature by revealing three new findings. First, an inverse association was identified between psychological resilience and IGD among Chinese adolescents. The degree of depressive symptoms was positively associated with both IGD and risky online behaviour. Second, the results highlight the mediating role of psychological resilience in the association of depressive symptoms with both IGD and risky online behaviour. Finally, the hypothesised moderating role of sex was not significant, indicating that sex is not a necessary factor to consider when designing intervention programmes aimed at mitigating the risks of IGD and risky online behaviour.

As predicted, psychological resilience was inversely associated with IGD. It is plausible that Chinese adolescents characterised by a lower level of psychological resilience may adopt less effective strategies for handling life stress, including academic and interpersonal problems (72). As a result, these adolescents may turn to online gaming to gain a sense of achievement and enjoyment, as well as to form connexions with others in the cyber world (73). According to psychodynamic perspectives, the relationship with the primary caregiver is a significant factor influencing the development of resilience (74). Emotional regulation and the capacity to cope have been shown to be associated with psychological resilience, which mediates the association between attachment security and overall well-being, further influencing interpersonal relatedness and adaptation across the lifespan. Adolescents with a higher level of psychological resilience tend to have a greater tolerance for unpleasant emotions and greater family support to deal with life challenges (49, 75). Likewise, the same explanation may also be applied to the criterion of risky online behaviour. Taken together, a poor child–caregiver relationship is associated with a high dependency on online activities and exposure to high online risks (76, 77). Individuals experiencing depressive symptoms with difficulties in emotional regulation and interpersonal relationships tend to use online social interactions, such as online chats and gaming with strangers, as coping strategies (78, 79).

This study unveils the processes underlying IGD and risky online behaviour. Psychological resilience accounts for the association of depression with both IGD and risky online behaviour. Such associations between IGD and mental health may be due to social isolation resulting from spending too much time gaming, which compromises psychological well-being (79, 80). However, psychological resilience serves as a protective factor between depression and IGD symptoms, plays a role in depression, and fosters effective coping in those with depression (5). Adolescents with a higher level of psychological resilience tend to use a variety of resources to cope with depression and mitigate IGD and risky online behaviour. Such an interpretation is consistent with the conceptualisation of addiction, as maladaptive coping strategies in handling adverse psychological problems tend to promote escapism. As the Internet has been regarded as a refuge to avoid coping with real-life problems (81), individuals with depression turn to online games to deal with their emotional problems (38). However, excessive use of this avoidant coping strategy via online gaming may leave real-life problems unresolved, thus eliciting psychosocial maladjustment and increasing depressive symptoms in the long term. It is worth considering that IGD and depression may share similar risk factors, such as education level, psychosocial environment, coping skills, and family background. All of these factors may exacerbate the severity of both IGD and depressive symptoms.

Contrary to our predictions, the hypothesised moderation effect of sex on the mediation model was non-significant. The adolescents in our study may have been too young to show sex differences in psychological resilience. Such differences are fostered through personal development and environmental factors, to which individuals are exposed over the course of their lives. This notion stems from previous work revealing sex differences in psychological resilience among older participants (82, 83). Nevertheless, the findings of this study demonstrate that male adolescents tend to report more frequent exposure to violent content online and cyberpornography than their female counterparts. Moreover, male adolescents tend to be at higher risk of IGD and risky online behaviour than their female counterparts. Our findings are in line with previous findings (84), which indicate that male online gamers tend to keep to themselves, increasing problematic Internet use. Moreover, our analysis revealed that female adolescents more commonly experience depressive symptoms, which is consistent with a myriad of studies (see for a review) (85). Although women are more sensitive than men to the adverse effects of mood disturbance, they tend to adopt more effective strategies of coping with their emotional problems (86) and engage more in emotional expression (87). Hence, female adolescents are less likely to use online gaming as a coping strategy than male adolescents. Furthermore, this study showed no sex difference in the level of psychological resilience, a finding that is consistent with previous empirical evidence (36).

Many studies have adopted a symptom approach that focuses exclusively on adolescents' psychological problems and their associations with IGD and online risky behaviour (88, 89). A major theoretical contribution of this study is that it adopted a positive, immunisation approach that evaluates the beneficial role of psychological resilience on IGD and risky online behaviour. Adopting a positive, immunisation approach may have practical implications for the design of intervention programmes to prevent these emergent problems in the cyber era.

For mental health practitioners and teachers, our new findings may invigorate interest in psychological resilience as a core element in preventive interventions for IGD and risky online behaviour. To support adolescents who experience adversity, parents, clinicians, and teachers may focus on building adolescents' strengths and capabilities in managing online risks instead of focusing on their problems and weaknesses. Intervention programmes, including psychoeducation, discussion, and group work, should strengthen some major protective factors (e.g., psychological resilience) to mitigate unpleasant emotions derived from stressful real-life encounters. To reach these goals, some important steps to build resilience should be involved, namely effective coping strategies to handle real-life personal and interpersonal problems and training on emotional regulation and life skills (e.g., goal setting and communication). An additional benefit of including psychological resilience in school programmes is a mitigated risk of problematic Internet use and online gaming by students. Another implication of this research is that schools, teachers, and social workers should promote discipline to prevent the risk of IGD among adolescents and the deterioration of daily life functioning among those with IGD.

To conclude, this study extends the literature by demonstrating the beneficial role of psychological resilience as a protective factor that reduces the likelihood of IGD and risky online behaviour. Chinese male adolescents tend to have a higher tendency for IGD, risky online behaviour, and exposure to online violent content and cyberpornography compared with female adolescents. Overall, the findings of this study demonstrate that psychological resilience should be prioritised in the prevention and treatment of IGD and risky online behaviour. Additional efforts are needed to strengthen psychological resilience and well-being among Chinese adolescents, especially those with high exposure to gaming and other risks on the Internet.

## Data Availability Statement

The original contributions presented in the study are included in the article, further inquiries can be directed to the corresponding author.

## Ethics Statement

The studies involving human participants were reviewed and approved by the Human Research Ethics Committee of the University of Hong Kong. Written informed consent to participate in this study was provided by the participants' legal guardian/next of kin.

## Author Contributions

YT and CC contributed to the conception, design of the study, and conducted the literature review. YT coordinated the data collection process, performed the statistical analysis, and wrote the first draft of the manuscript. CC wrote sections of the manuscript. Both authors contributed to the editing, revision of the manuscript, and approved the submitted version.

## Funding

This study was funded by Hong Kong Research Grants Council's General Research Fund (grant number 17400714) and the University of Hong Kong's Seed Fund for Basic Research (grant number 201711159216) to CC.

## Conflict of Interest

The authors declare that the research was conducted in the absence of any commercial or financial relationships that could be construed as a potential conflict of interest.

## Publisher's Note

All claims expressed in this article are solely those of the authors and do not necessarily represent those of their affiliated organizations, or those of the publisher, the editors and the reviewers. Any product that may be evaluated in this article, or claim that may be made by its manufacturer, is not guaranteed or endorsed by the publisher.
